# The comparison of the effects of neuromuscular electrical stimulation and Kinesio Taping on ankle swelling in athletes with lateral ankle sprain

**DOI:** 10.1186/s40634-023-00624-w

**Published:** 2023-06-10

**Authors:** Vahid Mazloum, Hadi Akbari, Anis Gholampour

**Affiliations:** 1 Clinical Care and Health Promotion Research Center, Karaj Branch, Islamic Azad University, Karaj, Iran; 2 Department of Sport Sciences, Faculty of Literature and Humanities, University of Zabol, Zabol, Iran; 3 Faculty of Physical Education and Sports Sciences, Karaj Branch, Islamic Azad University, Karaj, Iran

**Keywords:** Ankle injuries, Lymphatic system, Edema, Electrode, Elastic taping

## Abstract

**Purpose:**

Ankle swelling (AS) is one of the main complaints in athletes with a lateral ankle sprain (LAS) in the acute phase. Reducing AS may help the athlete to return to training faster. The purpose of this study was to evaluate the efficacy of Kinesio Taping® (KT) and neuromuscular electrical stimulation (NMES) in reducing AS in athletes with a LAS.

**Methods:**

Thirty-one athletes with a unilateral ankle sprain from various sports were allocated to either KT (*N* = 16; mean age of 24.1 years) or NMES (*N* = 15; mean age of 26.4 years) groups. KT was applied over the medial and lateral ankle surfaces in the Fan cut pattern for five consecutive days; however, NMES was applied to the tibialis anterior and gastrocnemius muscles for 30 min. Outcome measures to assess the extent of AS included volumetry, perimetry, relative volumetry, and the difference in both ankles’ volumetry and perimetry at baseline, after the interventions, and 15 days following the treatment completion.

**Results:**

The results of the mixed model repeated measures ANOVA demonstrated no significant difference between the two groups in mean changes in outcomes over pre- and post-interventions as well as follow-up periods (*P* > 0.05).

**Conclusions:**

None of the KT and NMES methods could reduce acute AS in athletes with LAS. Further studies are needed in this area of research that consider changes in treatment protocol given the variety of NMES approaches and KT applications that can be used in recovery from an ankle sprain.

## Background

Ankle sprains are one of the most common sports injuries occurring in various sports [1, 2]. In high-performance athletes, ankle sprains can lead to significant performance limitations as well as economic problems due to absenteeism [3]. The main complaints of acute ankle sprains include pain and swelling [3, 4]. If not properly treated in the acute phase, this injury can progress to other injuries such as synovitis, tendinopathy, joint stiffness, muscle weakness, joint instability, and persistent pain and swelling [1, 3, 4]. After ankle sprains, about 60% of people have symptoms for up to 18 months after the injury, which increases the likelihood of recurrence [5]. Among the complaints of ankle sprains, swelling requires careful and immediate attention because it is associated with the progression of inflammation and can limit the rehabilitation process [3, 5].

The standard treatment plan to control swelling in ankle sprains includes rest, ice, compression, and elevation, which is called the RICE protocol [6]. In addition, physiotherapists may apply other interventions to minimize swelling. Electrical stimulation with or without muscle contraction is one of these interventions [7]. Neuromuscular electrical stimulation (NMES) causes muscle contraction, which can facilitate drainage of the veins and lymphatic vessels. This mechanical effect may help eliminate or reduce edema after trauma [8]. Frequent contractions may lead to increased venous return [9] and lymphatic flow [5], which in turn reduces edema [5, 7, 8]. Few studies have examined the direct effect of motor electrical stimulation on reducing swelling through evaluating the alteration in swelling [10]. To our knowledge, there are no studies that have investigated the direct effect of motor electrical stimulation on ankle swelling (AS).

Another modern method used to reduce swelling is Kinesio Taping® (KT), which is popular with physiotherapists and rehabilitation specialists. This technique involves the application of an elastic tape with a weight and thickness similar to the parameters of human skin directly to the skin. KT does not restrict movement and is waterproof and breathable due to its wavy texture [11]. KT is well used in chronic ankle instability, which may occur after repetitive sprains [12]. Some believe that applying this method to the ankle stimulates the drainage of edema in the interstitial tissues to areas with a lower density of lymphatic vessels, which ultimately leads to a reduction in swelling [11]. Some studies have reported positive results of using KT compared with placebo taping or other manual therapies to treat swelling (such as manual lymphatic drainage) [13–15]. Regarding the results derived from the very few available studies, particularly in the field of AS related to lateral ankle sprain (LAS), the effect of KT on acute AS remains in doubt.

Therefore, this study aimed to compare the respective effects and sustainability of KT and motor electrical stimulation on AS in athletes.

## Methods

The present study was a randomized clinical trial. A priori power analysis (G*Power for Windows, version 3.1.7) revealed that 15 participants per group were required to obtain 80% statistical power with a medium effect size (f = 0.24) [16] and an alpha of 0.05. To account for possible missing data, 35 male semi-professional athletes in 2022 were included in this study. They were employed by their clubs, but not full-time; they were underpaid, so they could not live adequately on that alone. These athletes had unilateral ankle sprains within 48 to 96 h before initial evaluation with visible AS. An experienced orthopedic surgeon conducted the initial evaluation. Participants were excluded from the study if they had: fractures; open wounds; lower extremities edema in systemic diseases like heart, renal or venous diseases; allergic response to kinesio tape; the presence of a metal piece in the ankle bones, injury to the opposite ankle that required surgery over the past year, taking blood pressure medications; or a cardiac pacemaker. They were randomly assigned into NMES (*n* = 18) and KT (*n* = 17) groups.

In this study, the procedures in each group were performed on 5 consecutive days. A senior physical therapist with more than 15-year experience performed the interventions for NMES and KT groups. NMES was performed over the tibialis anterior and gastrocnemius muscles for 30 min per session. However, KT was applied to the medial and lateral surfaces of the ankle. Outcome measures to evaluate the extent of AS included volumetry, perimetry, relative volumetry, and difference in both ankles volumetry and perimetry evaluated at baseline, after receiving the interventions, and 15 days following the treatment completion.

To evaluate the extent of AS, the method of volumetry for the healthy and injured sides was used, which was used for the same purpose in a previous study with intra-rater and inter-rater reliabilities of 0.98 and 0.99, respectively [17]. For this purpose, the volume of water, which was proportional to the volume of the foot, was slowly placed on a digital scale (with 1-g accuracy). The collected data were converted to milliliters (assuming that each gram corresponds to one millimeter) [18]. The order of assessment of the injured and healthy ankle was randomized for each subject.

Ankle perimetry was measured with a tape based on the figure of 8 (ankle circumference using eight ankle/foot landmarks). The subject was in the supine position and the ankle was positioned in the neutral eversion-inversion position in 90° dorsiflexion. The landmarks were: Tibialis anterior tendon, tuberosity of the navicular, base of the 5th metatarsal, again Tibialis anterior tendon, medial malleolus, Achilles tendon, lateral malleolus, and again Tibialis anterior tendon. The landmarks must be connected in the shape of figure eight. Each leg was examined three times and the order of foot selection was random. The mean values of the three measurements were recorded. The intra-rater and inter-rater reliability for this procedure was reported 0.98 and 0.99, respectively [17].

**Fig. 1:**
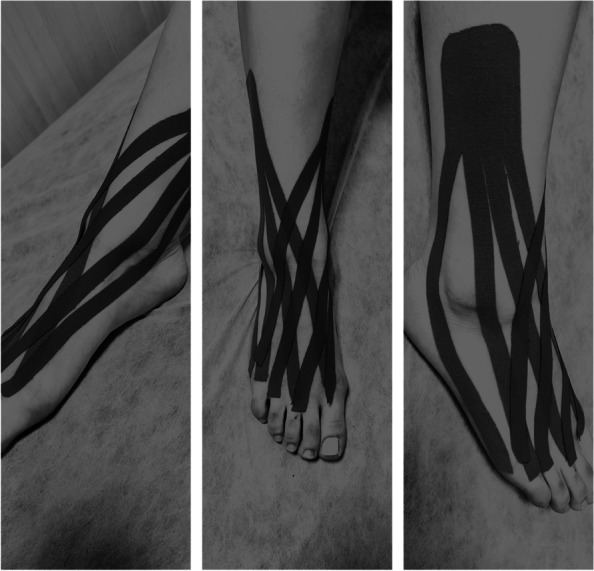
Full KT application with the fan cut pattern

The intervention was performed in the KT group using Tem Tex Kinesio Tape (5 cm wide and 5 mm thick) as previously reported [11]. The day before the KT application, the subject was asked to shave the area. KT was used with the fan cut pattern (Fig. 1). The subject was placed in a supine position and was marked 13 cm above the lateral malleolus and 10 cm above the medial malleolus of the injured ankle. The subject was then asked to move his foot to the plantar flexion and 5 degrees of inversion so that the required tape could be measured and cut (the distance between the top point of the lateral malleolus and the fifth toe was measured). The other tape, which was placed on the medial surface of the ankle, was cut to the size of the previous tape. KT tapes on the medial and lateral surfaces of the ankle started from the marked points and continued to the metatarsals of the foot with 20% tension [11]. The KT tape was made into four narrower tapes, each of which was applied at a distance of about 1 cm from the adjacent tape. The tape of the lateral surface was applied in the direction of the fibula bone, and each of the four tapes was applied to these sections in sequence: behind the lateral malleolus, on the lateral malleolus, in front of the lateral malleolus, and the direction of the thumb [11]. The KT tape of the medial surface was slightly inclined to the tibia, and the arrangement of the tapes was as follows: behind the medial malleolus, on the medial malleolus, in front of the medial malleolus, and the direction of the little finger. The part of the tape that was not detached was placed on the skin in the neutral position of the ankle, while the position of the ankle when applying four narrower tapes was maximally plantar flexion and about 5 degrees of inversion.

**Fig. 2:**
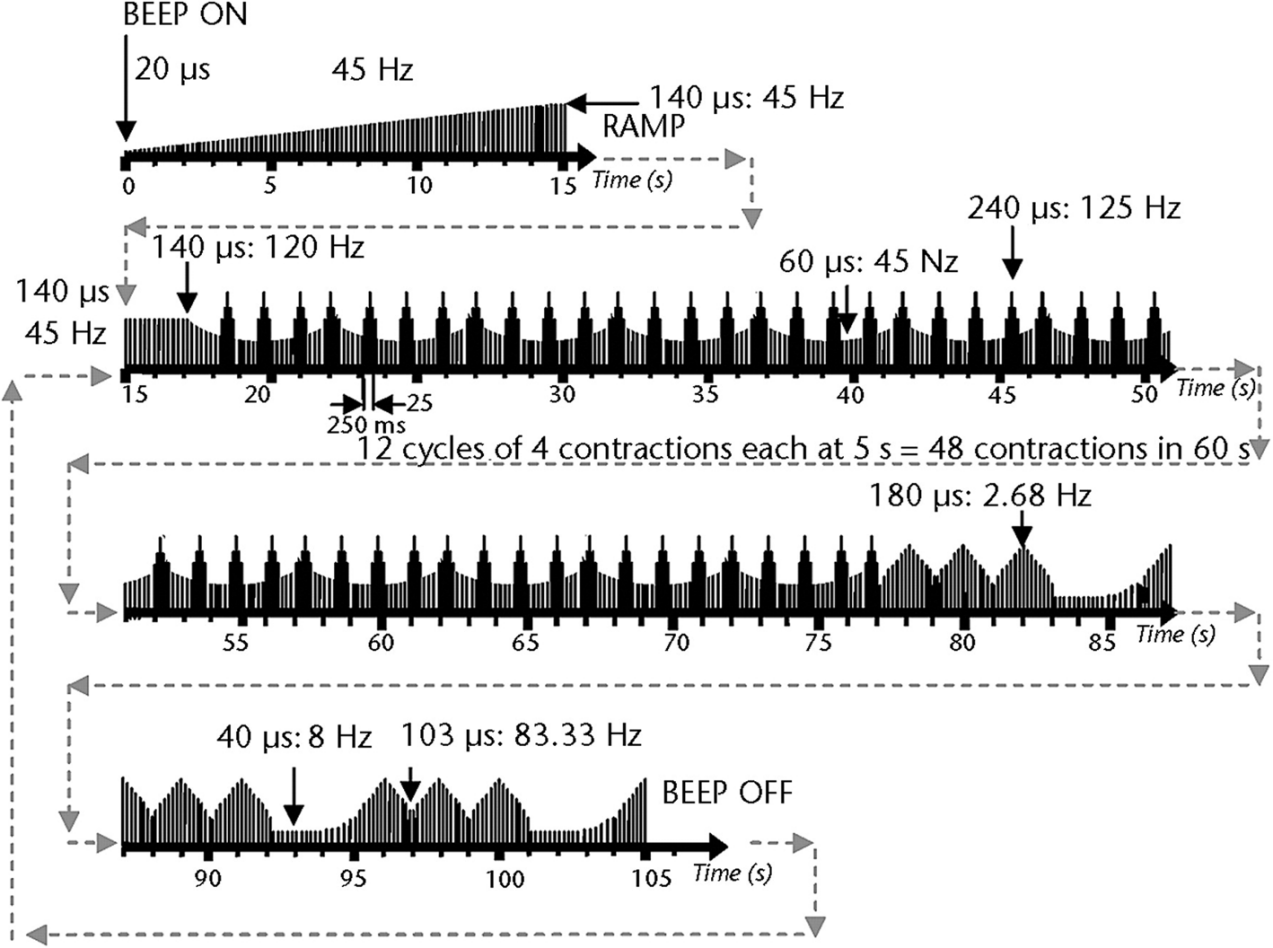
Diagram of electrical stimulation pattern with a low-voltage, rectangular waveform having modulated patterns of frequency and pulse duration, adopted from Man et al. [20]

In the NMES group, the HEALTHFIT electrical stimulation device (dual-channel P4-Microstim stimulator) with four electrodes was used. The patient was placed in the supine position, the injured leg was elevated above the level of the heart and placed on a chair and fixed with a strong elastic band. To minimize any movement of the ankle, the sole was placed against the wall. The electrode used in this study was a 10-cm-thick Carbonflex disk made of conductive carbon rubber and covered with a sponge pad to avoid direct contact with the skin. Two electrodes were placed on the bulk of the gastrocnemius muscle and two electrodes were placed on the tibialis anterior muscle. Electrical stimulation was applied with a low voltage and a rectangular waveform and modulation of frequency and pulse duration (Fig. 2). The duration of NMES was 30 min (360 cycles of 5 s each). Each cycle consisted of 400 pulses with different combinations of pulse-to-pulse time intervals and duration values [19, 20]. The average frequency of electrical stimulation was calculated at 80 Hz, but electrical stimulation was received in the form of bursts that elicited short-term muscle contractions every 1.25 s (0.8 Hz). During each burst, the pulse duration varied from 60 to 240 ms, and the pulse-to-pulse time intervals were 8 ms (125 Hz). During this burst pattern, there was another predetermined pattern of electrical stimulation. This pattern had a 5-s backward cycle and a pulse frequency of 45 to 120 Hz with a concomitant change in pulse duration of 60 to 140 microseconds. The last phase of the cycle began 77 s after the start of the device. The frequency range was from 2.68 to 83.33 Hz and the pulse duration was from 40 to 180 microseconds [19, 20]. The intensity of the applied electrical stimulation was increased to the maximum tolerance of the individual [20] and changed over 30 min if necessary.

The absolute score of the injured ankle was used for the first volumetry analysis. The next volumetry analysis was performed as a percentage of body mass (in grams) (ankle volume/mass × 100) and was referred to as relative volumetry. In the next analysis, the raw perimetry data of the injured ankle were used and the difference between the volumetry and perimetry of the healthy and injured ankle was calculated.

The dependent variables were analyzed in three phases: before the intervention, after the intervention, and 15 days after the completion of the intervention. Data were analyzed using SPSS, version 22. A 3 × 2 repeated measures ANOVA was used to compare the effect of time (pre-test, post-test, and follow-up) and group (KT and NMES) on the dependent variables between groups. Furthermore, the Shapiro–Wilk test was employed for assessing the normality of the distribution of scores. Levene’s test was used to assess the homogeneity of variance between groups. A significance level was considered at the 95% confidence level for all statistical parameters.

## Results

Since four subjects did not complete the study, statistical analysis was performed on 31 subjects (15 for NMES and 16 for KT) who participated in pre-intervention, post-intervention, and follow-up measurements. The characteristics and demographic factors of the participants are presented in Table 1. There was no significant difference between groups in the characteristics of participants at baseline (*P* > 0.05). The mean values (± SD) of the dependent variables of the two groups before the intervention, after the intervention, and at follow-up are presented in Table 2.

**Table 1:** Male athletes’ characteristics (Mean ± SD) among groups

	**NMES Group**	**KT Group**	***P*-Value**
Age (y)	26.3 ± 4.8	24.4 ± $1.3$	0.139
Weight (kg)	79.8 ± 2.1	81.6 ± 9.1	0.287
Height (m)	1.79 ± 0.09	1.83 ± 0.05	0.227
BMI (kg/m2)	24.0 ± 4.6	24.0 ± 4.7	0.838
Duration after injury (h)	62.9 ± 8.6	70.12 ± 8.3	0.071

*y* Year, *kg* Kilogram, *m* Meter, *kg/m2* Kilogram per square meter, *h* Hour, *p*-value = independent t-test value among groups

**Table 2:** Dependent variables measure (Mean ± SD) over three times phases among groups

Variable	Pre-intervention		Post-intervention		Follow-up (15 days later)	
	**NMES Group**	**KT Group**	**NMES Group**	**KT Group**	**NMES Group**	**KT Group**
Volumetry (ml)	1583.35 ± 31.65	1584.13 ± 30.51	1583.85 ± 22.34	1583.31 ± 24.57	1584.11 ± 24.09	1582.62 ± 29.48
Perimetry (cm)	53.16 ± 5.36	54.81 ± 4.58	54.29 ± 6.14	56.55 ± 4.33	52.19 ± 3.41	53.42 ± 3.82
Relative volumetry (percent)	0.55 ± 2.17	0.47 ± 4.85	0.09 ± 10.96	1.02 ± 9.21	2.49 ± 6.13	1.31 ± 4.35
Difference in both ankles volumetry (ml)	53.64 ± 6.97	56.12 ± 4.97	51.26 ± 8.61	53.84 ± 3.65	51.85 ± 6.21	51.47 ± 5.44
Difference in both ankles perimetry (cm)	-0.97 ± 3.61	0.20 ± 3.90	1.85 ± 2.90	0.66 ± 2.83	1.16 ± 2.62	0.63 ± 3.41

*ml* Milliliter, *cm* Centimeter

The results of mixed model repeated measures ANOVA failed to identify the main effects for any of our variables between the intervention groups as well as periods and no group × trial interaction effect (*P* > 0.05).

## Discussion

In this study, two conservative interventions—NMES and KT—were used to reduce AS in athletes with ankle sprains. The results failed to demonstrate any statistically significant changes in volumetry, perimetry, relative volumetry, the difference in volumetry, and perimetry of either the injured or healthy ankle and there were no statistically significant differences between the two groups after the intervention and a 15-day follow-up.

Our results showed that KT application did not significantly change ankle swelling in athletes with acute ankle sprains. The study has caused consistent results with those of Nunes et al. [21], who found KT application for 3 days is ineffective in decreasing acute swelling (measured via volumetry, perimetry, relative volumetry, and two analyses of the difference in volume and perimetry between ankles of each participant) after ankle sprains in athletes. However, our results are not consistent with those of Aguilar-Ferrándiz et al. [13], who found that the use of KT can reduce extracellular fluid in 156 individuals with chronic venous insufficiency. In conditions such as chronic venous insufficiency, swelling is expected to occur as a result of changes in hydrostatic pressure that lead to low levels of protein and transudate [22]. In our study, subjects had acute ankle sprain associated with an active inflammatory process and an increase in some inflammatory mediators and associated protein levels. Therefore, it is possible that the effect of the KT technique on swelling is limited to venous insufficiency [11, 13, 15] and is not sufficiently effective in the acute phase of swelling. Another reason could be that they used a Bioelectrical impedance meter with Skintact RT-34 adhesive electrodes and four connectors to measure and evaluate the extent of peripheral edema [21]. They recorded cell mass, intracellular and extracellular water, and fat mass. The initial formation of leg venous edema was considered an increase in extracellular water [21]. In our study, a mathematical convention that converted ankle perimetry size to ankle volumetry was used to calculate the extent of swelling reduction. Such contradictory results increase the likelihood of confirming the hypothesis that the effects of KT on swelling are limited to changes at the cellular level (reduced extracellular liquid from the lower limbs) of chronic venous disease and have no effect on the actual volumetry of this part (stimulating the lymphatic system), contrary to the beliefs of the founders of KT that this technique could stimulate the process of reabsorption of interstitial fluid through the lymphatic system [11, 13, 15].

It is recommended to continue the application of KT for 3 to 5 days until the strands do not lose their elastic properties [11]. In the present study, the maximum suggested time was considered the duration of the procedure (i.e. 5 days). Probably, one of the reasons for the ineffectiveness of this technique is related to the duration of application, because some studies have shown that the application of KT for 10 consecutive days resulted in a decrease in swelling [13, 14]. Yet, there was a possibility that for 10 days, the swelling decreased by itself, not due to KT application. Since they have no control group [14] the findings remain ambiguous. In the study by Aguilar-Ferrándiz et al. [13], the KT was applied 3 days per week for 4 weeks, which strengthens the hypothesis of the effect of the duration of the technique. However, because our study aimed to investigate the effect of KT on swelling in the acute phase of ankle sprain, the duration of the intervention was not extended. Another reason for the ineffectiveness of KT on swelling reduction in athletes with ankle sprain could be that the stimulation that KT exerts on the skin and lymphatic system is probably not strong enough to cause changes in the acute swelling.

Another intervention that the subjects in this study received was NMES. Our results in this regard consist with those of the study by Man et al. [20], in which NMES is not effective in decreasing ankle–foot volume. The NMES treatment strategy of our study and theirs were identical. It appears that the treatment strategy with NMES used in the study need to be modified or changed the method to achieve the desired effects. Our results contrast with those of the study by Io et al. [19]. In that study, the method of motor electrical stimulation used was found to be effective in limiting the increasing trend in ankle and foot volume that results from prolonged standing. The two studies differed from each other in terms of the subjects. The subjects who participated in our study had swelling due to a musculoskeletal injury (ankle sprains). Such an injury tends to cause swelling of an inflammatory nature, in which capillary permeability increases, leading to the accumulation of interstitial fluid and swelling [23]. On the other hand, swelling caused by prolonged immobility is venous and results from increased venous pressure. Since NMES reduces the excess fluid by stimulating the intramuscular pump (resulting in a decrease of the increased venous pressure). This mechanism can be considered the main factor in reducing swelling due to increased venous pressure and its ineffectiveness in swelling due to increased capillary permeability. Our results also contrast with those of the study by Wainwright et al. [24], in which edema (as measured by fluid displacement) was statistically significantly reduced in patients with ankle sprains who received the current standard of care plus NMES use compared with the current standard of care only. However, consistent with our results, they showed no differences in ankle perimetry (recording figure of eight values) between groups. The current standard of care included patient education, manual therapy when indicated, and personalized exercise prescriptions. The intervention group was trained to wear the device on their injured leg during waking hours each day. The device was worn for a minimum of eight and a maximum of 16 h per day, with NMES usage recorded in patient diaries. From the results of this study, it can be concluded that the effectiveness of NMES is likely to increase when it is combined with other therapeutic interventions. It has been advocated that NMES can induce contraction and theoretically compress venous and lymphatic vessels, which may contribute to the resolution of edema [25]. Considering that our results are based on a small sample of athletes from different sports and that there is no consensus on the protocols for use of neither in LAS, despite the value of the outcome measures used for assessing the extent of AS, the results of such analyzes should therefore be treated with considerable caution. The beneficial effects of this intervention could be observed in our study if the subjects receiving it were not in the recovery phase after ankle sprains or if the type of methods used to measure the variables were appropriate. The electrical stimulation parameters and variables used, and the duration of treatment should be different in future studies to probably observe the beneficial effects of the NMES intervention when it is used in isolation in reducing swelling. Our study indicates that neither intervention of NEMS and KT had a significant effect on reducing variables related to the extent of AS and did not have superiority over the other after the interventions and the 15-day follow-up period. Further studies are needed in this area of research that consider changes in treatment protocol given the variety of NMES approaches and KT applications that can be used in recovery from ankle sprains.

## Conclusions

Neither the KT nor the NMES demonstrated efficacy in reducing variables related to the extent of AS due to ankle ligament sprains (volumetry, perimetry, relative volumetry, difference in volumetry, and perimetry of both injured and healthy ankles).

## Data Availability

The datasets used and/or analyzed during the current study are available from the corresponding author upon reasonable request.
